# Using sentiment analysis to predict opinion inversion in Tweets of political communication

**DOI:** 10.1038/s41598-021-86510-w

**Published:** 2021-03-31

**Authors:** Yogev Matalon, Ofir Magdaci, Adam Almozlino, Dan Yamin

**Affiliations:** Industrial Engineering Department, Tel Aviv University, 69978 Tel Aviv, Israel

**Keywords:** Scientific data, Complex networks

## Abstract

Social media networks have become an essential tool for sharing information in political discourse. Recent studies examining opinion diffusion have highlighted that some users may invert a message's content before disseminating it, propagating a contrasting view relative to that of the original author. Using politically-oriented discourse related to Israel with focus on the Israeli–Palestinian conflict, we explored this Opinion Inversion (O.I.) phenomenon. From a corpus of approximately 716,000 relevant Tweets, we identified 7147 Source–Quote pairs. These Source–Quote pairs accounted for 69% of the total volume of the corpus. Using a Random Forest model based on the Natural Language Processing features of the Source text and user attributes, we could predict whether a Source will undergo O.I. upon retweet with an ROC-AUC of 0.83. We found that roughly 80% of the factors that explain O.I. are associated with the original message's sentiment towards the conflict. In addition, we identified pairs comprised of Quotes related to the domain while their Sources were unrelated to the domain. These Quotes, which accounted for 14% of the Source–Quote pairs, maintained similar sentiment levels as the Source. Our case study underscores that O.I. plays an important role in political communication on social media. Nevertheless, O.I. can be predicted in advance using simple artificial intelligence tools and that prediction might be used to optimize content propagation.

## Introduction

Social media networks have become a vital tool for sharing information and for influencing opinions and decision-making^1–3^. Furthermore, the impact of social media on political discourse is growing^4^. It enables institutions and citizens to directly interact with each other, allowing more direct and active involvement in political decision-making processes^5^. In addition, social media platforms have proven to be highly influential in recent political events, such as the 2008 and 2016 U.S. presidential elections^6–8^, and the Arab Spring in the early 2010s^9^.

Thanks to its attractive and straightforward platform of over 300 million monthly active users as of 2019, Twitter has become one of the most influential social media networks^10–12^. Twitter has emerged as one of the most influential social media platforms in the realm of political discourse. By analyzing Twitter data alone, previous studies were able to predict election results^13,14^, identify homophiles and political ties in social networks^15,16^, and identify communication patterns and social interactions of political events^6,17^. In addition, interventions via Twitter were shown to be highly influential in political activity^18,19^. For example, automated Bots^18^ and the intentional spread of disinformation, commonly referred to as "fake news", were shown to negatively affect political discussion and endanger the integrity of elections^19^.

Political discourse concerning Israel is exceptionally active, attracting strong emotions, driving engagement on social media, and significantly impacting real-world events. In particular, discourse on Israel has spread outside the domain of politics to encourage the boycott of Israeli products, companies^20^, and various events, such as the 2019 Eurovision Song Contest^21^ and an Israel–Argentina football match^22,23^. In some cases, debate participants use non-political content to increase or decrease support for Israel.

The observation that social media has a major impact on political settings outcomes triggered multiple studies to explore how to increase Tweets' propagation^24–28^. By analyzing 74 million Tweets, Suh et al.^25^ showed that URLs and hashtags in a Tweet are the strongest drivers of retweet rate, which is a crucial measurement to infer the overall propagation of a message in the network. Nam et al.^24^ also found that groups of Tweets related to a particular keyword or topic have distinctive diffusion patterns and speeds related to the Tweet's content characteristics when being retweeted. More recently, DePaula et al.^26^ found that Twitter user engagement in local government in the U.S. is closely associated with symbolic and image-based content. These studies underscored that a message's content is at the core of user engagement.

Other factors that influence user engagement with a Tweet may be unrelated to the message's actual content and can be analyzed using automated means. For example, analyzing the emotionality and sentiment of a message yields new signals that are highly indicative of whether a message will spread and engage users^29,30^. Berger et al.^31^ examined the link between message emotion and virality for nearly 7000 emailed New York Times articles. The authors demonstrated that articles that evoke high-arousal emotions, such as awe, anger, and anxiety, are more viral than articles that evoke low-arousal emotions such as sadness. Hansen et al.^32^ found that news with negative sentiment was more viral than news with positive sentiment. In addition, the sentiment of political Tweets can be used to track and impact political opinions^33^, to detect consistency between the stated and actual preferences of politicians, and to predict election results^13,34,35^. Thus, analysis of sentiment and emotions is at the center of social media research, serving as a powerful content framing tool for increasing virality^36^.

Accounting for the content of a message to evaluate exposure is particularly important in political debates because a response may invert the meaning of the original message (the Source) before sharing it, causing a negative outcome from the perspective of the original author^37,38^. Thus, to correctly measure total engagement with specific content within a social network, it is essential to explicitly weigh both the positive effects of engagements that agree with the Source and the negative effects of engagements that disagree with the Source.

The Twitter platform provides a straightforward way to assess the opinion of a user towards content. In April 2015, Twitter launched the "Quote" feature, which allows a user to retweet an original message with a comment. Using this feature, users can agree with, disagree with, or simply communicate the existence of a message. Garimella et al.^37^ found that the feature has increased political discourse and diffusion compared to existing features. By comparing the text of the comments accompanying "Quote" retweets to the original Tweet, they found a change between the "Quote" comment and the original text, with 4% of Quote texts disagreeing with the Source text. Guerra et al.^38^ found that social groups that hold views antagonistic to one another may retweet messages of antagonist groups more often than they retweet messages from other groups. Additionally, they underscored that retweets could carry a negative polarity, conveying a sentiment that is contrasting the view relative to the original author.

Here we developed machine-learning models to predict whether a Tweet will undergo Opinion Inversion, defined as a non-identical sentiment polarity between a Quote and its Source text (O.I.). Using politically-oriented discourse relating to the Israeli–Palestinian conflict, we investigated the relations between Source and Quote sentiments towards Israel. We identified strategic types of Quotes to sources that were unrelated to the conflict. Given the high impact of polarization on political discourse, our work can be utilized to optimize content propagation.

## Methods

### Twitter dataset

We extracted a random sample of 715,894 English language Tweets that were posted between January 6, 2008 and February 12, 2018 and included a set of 30 general keywords or hashtags related to Israel with a focus on the Israeli–Palestinian conflict. These keywords and hashtags cover a wide variety of organizations, key personals, and terminologies that are directly or indirectly related to Israel. In addition, they were found to be popular on Google Trends, consistent with a previous study^10^, or widely used by newspapers and reports of organizations that support or oppose Israel (see S1, Data collection).

To refrain from a bias related to a different interpretation of what is constitutes political content, we defined a Tweet to be *relevant* if it included any content linked to Israel, excluding weather and sports terms. By manually labeling 5000 Tweets by 7 Israeli students, we developed a relevance classification model to identify whether a Tweet is, indeed, relevant (see S2, a Relevance classification model). For example, the hashtag "#SJP" may refer to the American actress Sarah Jessica Parker (i.e., not relevant) but may instead refer to Students for Justice in Palestine, which is relevant. To evaluate the labeling process, we used a kappa coefficient (Cohen, 1960). The kappa statistic value for 100 Tweets was 0.95. Our model reached an accuracy of 0.96 and ROC-AUC of 0.98 on the test set, suggesting that 89% of the 715,894 Tweets carried out were relevant.

### Sentiment toward Israel

For a relevant Tweet, we developed a model to evaluate the Tweet's sentiment polarity toward Israel. Each Tweet is classified by our model as neutral (0), opposing Israel (− 1) or supportive of Israel (+ 1). After removing the irrelevant Tweets from the 5000 samples, we remain with 4500 relevant Tweets as input to the model. By manually labeling those Tweets by 7 Israeli students, we obtained a kappa statistic value of 0.804 for 100 Tweets. In order to ensure our labeling process is not biased, we created a coding schema for the students who tagged the data (see S9, Labeling schema). This polarity model reached 79% accuracy and a weighted F1 score of 0.78 on the test set (see S3, Polarity toward Israel classification model).

We then calculated the general sentiment of each Tweet using VADER^39^ model of the Natural Language Toolkit (NLTK)^40^. This widely used open-source algorithm specifies a sentiment score in the range [−1,1]. There are several approaches for identifying the sentiment on a sentence level (such as LIWC^41^). However, VADER is preferred for our needs because it is sensitive to social media sentiment^42,43^ and can be adjusted easily to a specific domain. To obtain a continuous scale with regards to sentiment toward Israel, we calculated the product of a Tweet's Polarity toward Israel, as determined by our model, and the absolute value of the sentiment analysis algorithm. To differentiate between non-neutral Tweets toward Israel and Tweets with neutral sentiment toward a general subject (NLTK value), we set the value of the sentiment toward Israel to $0\pm \epsilon$ in case the Polarity was not neutral and the NLTK value was equal to zero.

Additionally, we have compared our results to SentiStrength^44^ method, which implements a state-of-the-art machine learning method in the context of Online Social Networks^45,46^ (see S8, sentiment methods' comparison). We randomly sampled 500 pairs (1000 Tweets) and manually tagged the sentiment group of each Tweet (strong oppose, weak oppose, neutral, weak support, and strong support). We found that the VADER method was more accurate, with an accuracy of 80.2% (Table S9).

### Opinion Inversion prediction model

We developed a model that predicts whether a Source will undergo O.I. by analyzing Source–Quote pairs. We defined that a Tweet undergoes O.I. if the sentiment polarity toward Israel of the Quote does not match that of its Source.

#### Source–Quote pairs

From our data set, we identified 7147 Quotes (defined as Tweets whose text ends with a link to another Tweet^37^). For example: "Yet another Palestinian denied the right to enter his homeland. #BDS https://t.co/XXX". We then extracted the original messages (the Sources) from all the identified Quotes, yielding 14,294 Tweets written by 7783 users. Only 5 Quotes were created from another Quote, and 973 Sources were not related to Israel, but their Quotes were. Our analysis focused on the 6174 relevant pairs.

Each Tweet's polarity toward Israel (Sources and Quotes) was determined using the sentiment polarity classification model. The model's label is binary: 1 for a non-identical sentiment polarity toward Israel between a Quote and its original text (O.I.), and 0 otherwise.

We randomly sampled 90% Tweets as a training set and 10% as a test set. To analyze the training set, we developed a group of prediction features.

#### Prediction features

Since no study has examined the factors that drive contradiction between a Quote and its Source for political content, we created features based on known virality predictors^25,26,29–33^ and based on Quote's factors^37^.

The 36 features for the O.I. prediction model are categorized into three groups: content-driven features of the Source, features related to the user's profile, and the Source user's previous activity. For the full list, see Table S7.

##### Content features

Before each Tweet's content features were determined, each Tweet went through a pre-processing pipeline, including slang correction, stop-word removal, and stemming (see S2.1 Pre-process). The first features were derived from the sentiment of a Tweet toward Israel. In addition, we created the following features from the text of the Tweet:

- *Basic features* Number of characters, number of tokens.
- *Hashtags and mentions features* Number of mentions and hashtags in the Tweet.
- *Tweet content and media* Boolean features indicating whether the Tweet has a link or a photo embedded in it.
- *Emotions* we utilized the IBM Watson Tone Analyzer service^47^, in order to measure, for each Tweet, 13 emotion- and emotion-related characteristics: anger, disgust, fear, joy, sadness, analytical, confident, tentative, openness, extraversion, agreeableness, and emotional range.

##### User profile features

- *User bio* We analyzed the user description as presented on the user profile page. Taking the bag-of-words approach^48^, we searched the descriptions for keywords that may indicate a user's attributes.
- *User profile metadata* User features that were extracted from Twitter during data collection, including the number of followers, number of friends and whether the user is verified by Twitter.

##### User activity features

User activity information that was extracted from Twitter during the data collection, such as number of Likes and number of statuses.

### Model prediction

For feature selection, we considered both independent factors and the effects of interactions between all potential features. Using feature importance determined by a Random Forest model^49^, we removed features with an importance lower than 1%.

We considered four prediction models: Logistic Regression, Artificial Neural Network, Random Forest, and XGBoost:

- Logistic Regression^50^. The data were scaled using Z-standardization. Parameters were chosen to maximize the AUC. The regularization parameter was set to 0.1 with mean square error as a loss function, and using the liblinear solver.
- Artificial Neural Network^51^. We used a grid search with fivefold cross validation to select the structure of the network, the activation function, and the learning rate parameter. The final network was generated by batch gradient with 2 hidden layers, 50 nodes in each layer, logistic as an activation function and learning rate equal to 0.01.
- Random Forest^49^. We used an ensemble learning method that constructs multiple decision trees in a random subspace of the feature space. For each subspace, the unpruned tree generates their classifications, and in the final step, all the decisions generated by the number of trees are combined for a final prediction^52^. We performed a grid search with fivefold cross validation to select the number of trees, their depth, and the feature selection criteria. The final model contained 500 trees with Gini impurity criterion and a maximum depth of 5 in each tree.
- XGBoost^53^. XGBoost is a Scalable Tree Boosting System that can solve real-world scale problems using a minimal amount of resources. We performed grid search with fivefold cross validation to select the number of trees, the learning rate, the sampling ratios, etc. The model was trained to maximize AUC. The final model contained a 'dart' booster with 50 estimators where the learning rate was set to 0.01, maximum depth of 6 and subsample of 0.85.

**Figure 1:**
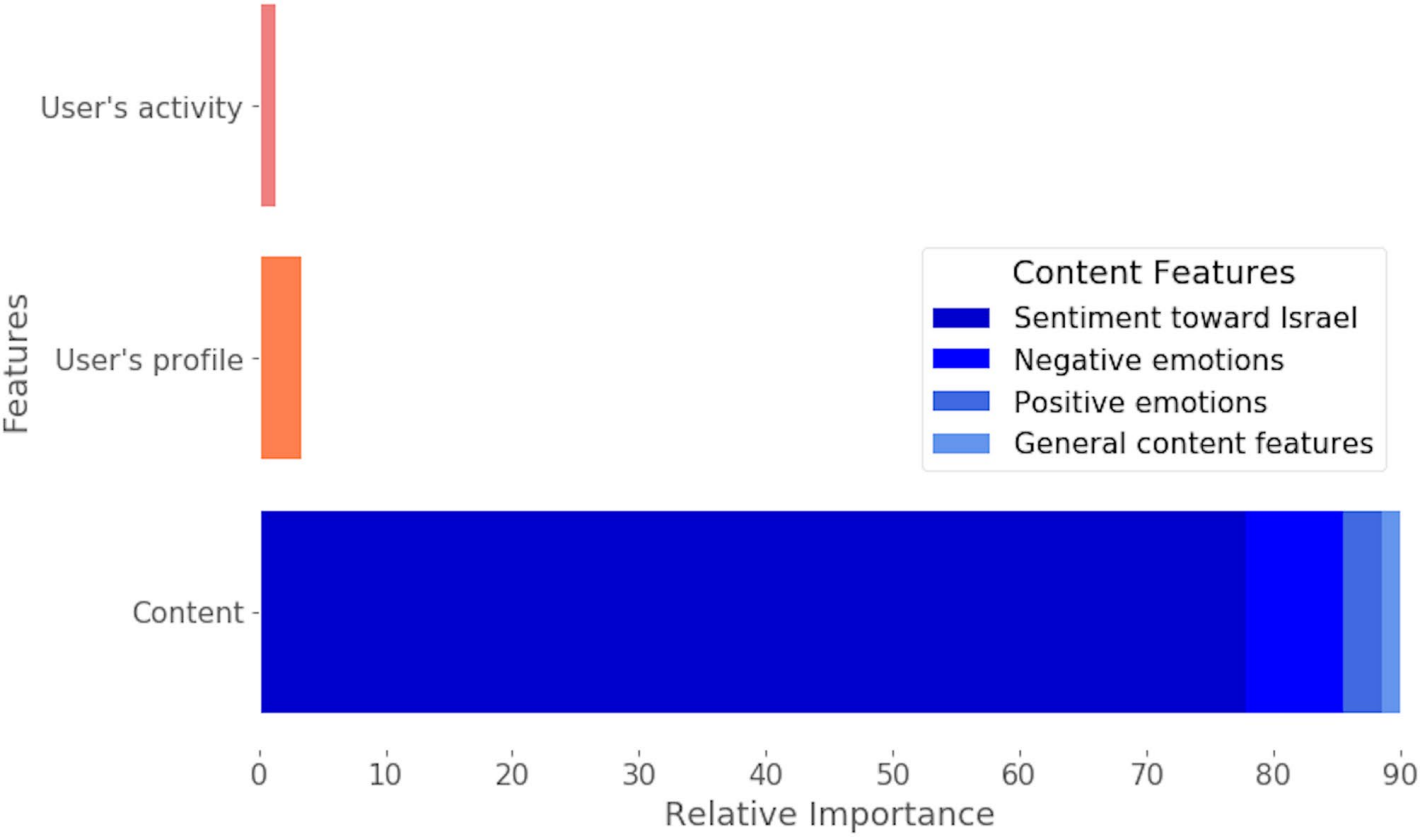
Aggregated feature importance of the O.I. prediction model. Relative importance was calculated by averaging the decrease in impurity over trees, considering the interaction between all the features. Aggregated features are subdivided into content, user profile, and previous user activity. Data shown were generated using the Random Forest model.

We examined each model by its ROC AUC result, accuracy, and F1 score on the test set (Table S6). The analysis of factors that explain O.I. (Fig. 1) contained an aggregated feature importance by content features (i.e., Polarity, sentiment toward Israel, sentiment group, and emotions) as well as user profile and user activity features. The non-aggregated feature importance is described in Figure S3.

### Sentiment dynamics analysis

Using each Tweet's sentiment, we classified Sources into five different sentiment groups: strong oppose, weak oppose, neutral, weak support, and strong support. Then, by examining the equality of Quote sentiment's distribution between each group and another group using the Kolmogorov–Smirnov test^54^, we found that the sentiment groups were significantly different with *p* value < 0.05. We then grouped all pairs whose Source sentiment falls into a particular range combination into a common set and calculated the average of the Source's sentiment and the Quote's sentiment separately for O.I. cases and for non-OI cases (Fig. 3a). We conducted the same process for the 973 irrelevant pairs that, although their sources were unrelated to Israel, their quotes were. Since the Source is irrelevant to our domain, we used the general sentiment (NLTK value) for the analysis of the Source, and the sentiment toward Israel for the Quote (Fig. 3c).

## Results

### Opinion inversion phenomenon

We analyzed a corpus of 715,894 English-language Tweets related to the Israeli–Palestinian conflict, and originally posted by 260,000 Twitter users between 2008 and 2018. By identifying 7147 Quotes, we found that while approximately 551,000 of the full corpus's Tweets had no Likes or Retweets, 4001 of the Quotes had at least one Retweet or Like. We then matched each Quote Tweet to its Source Tweet; these Source–Quote pairs corresponded to 69% of the corpus' total volume, defined as the total number of Likes and Retweets.

By developing a polarity classification model toward Israel, we classified each Tweet into three categories: Supportive, Neutral, and Opposing. For example, the Supportive category includes Tweets that revealed sympathy to Israel or opposed the other side. Using this classification, 66% of these Tweets showed antagonism towards Israel (Opposing), 15% showed sympathy toward Israel (Supportive), and the remaining 19% did not take any stand (Neutral) (Table S5).

We then examined changes in Polarity between Source and Quote. We defined that a Tweet undergoes O.I. if the sentiment polarity toward Israel of the Quote does not match that of its Source. For example, a Source with a Supportive Polarity toward Israel triggered a Quote with an Opposing or a Neutral Polarity toward Israel (Table 1). We identified that as many as 41% of Quotes inverted the opinion of the Source. In 33% of the O.I. cases, the Quote contradicted the Source text (i.e., transformed from a Supportive Polarity to an Opposing Polarity or vice versa). In 49% of the cases, the Quote took a non-neutral stand after engaging with a neutral Source, and in the remaining 18%, Quote text expressed a neutral polarity toward Israel, while the Source text expressed a non-neutral polarity.

**Table 1:** Examples of Sources and Quote-retweets, and their Polarity about the example domain.

	Source text	Source polarity towards Israel	Quote text	Quote polarity towards Israel	Opinion inversion (+/−)
1	Israel continues to take money from Palestinians while simultaneously continuing to demolish their homes #BDS	Opposing	Zionism is a disease that will suck the life out of any nation properly infected with it. #Palestine #BDS	Opposing	−
2	Muslim Student Group Blocks Holocaust Education Proposal dld.bz/fjjvV	Neutral	Muslim Students Association and SJP: we insist on calling Jews Nazis, but education about actual Nazis is bad	Supportive	+
3	"Despite disagreement on #IranDeal, I'm confident US&Israel will deepen cooperation in yrs ahead to address challenges we'll face together."	Supportive	I seriously doubt it, this is the beginning of the end. #Zionism #apartheid regime in #Palestine is going down!	Opposing	+
4	Palestinian children reportedly pretend to execute IDF soldiers in foreign aid-funded schools https://t.co/xKiQOzeCrl	Supportive	Why would you invite Abbas to the White House, @realDonaldTrump @POTUS	Neutral	+

A Tweet will undergo Opinion Inversion when the sentiment polarity toward Israel of the Quote does not match that of its Source.

We next developed several models to predict which original Source Tweets will undergo O.I. The prediction model included the Source's content features, features related to the user's profile, and information about the user's previous activity. Features related to contents included content length, sentiment toward Israel, and binary variables indicating specific feelings such as joy, fear, and anger. Features related to the user's profile included the number of followers and friends and its description. Features related to previous user activity included the number of prior statuses and Likes.

The Random Forest algorithm achieved the best performance of the tested models, with an ROC-AUC of 0.835 on the test set and an F1 score of 0.82 (Table S6). Regardless of the selected model, we found that content-driven features, and particularly the features describing the sentiments of a Source toward Israel, contributed the most to the prediction, accounting for 80% of the information gained (Fig. 1).

**Figure 2:**
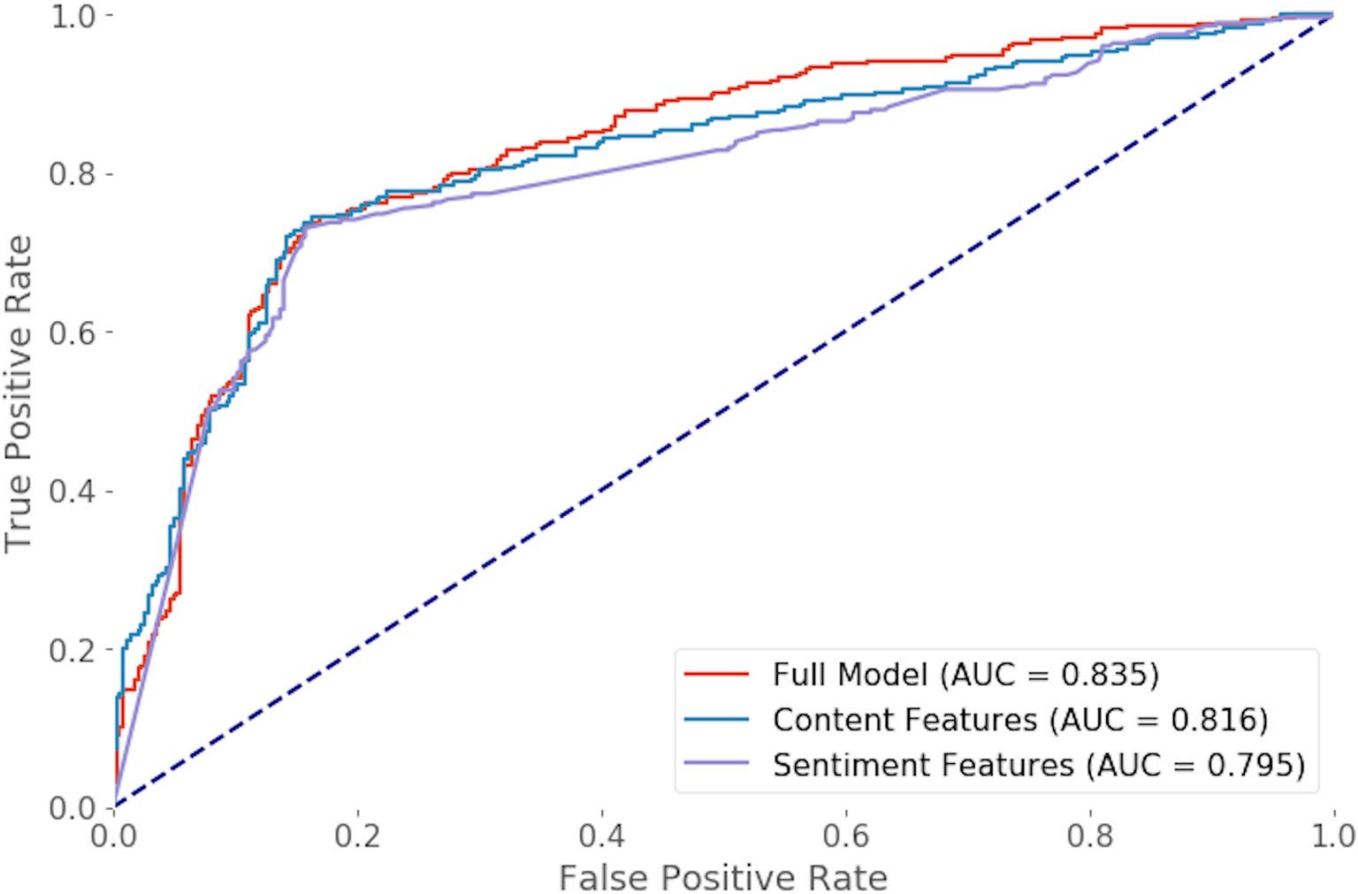
ROC curve of the O.I. prediction model. The legend indicates which features were used for the prediction.

Moreover, the model that only accounted for the sentiment features yielded an ROC-AUC of 0.795 (Fig. 2). A prediction model that included sentiment toward Israel, emotions, and features related to the content produced an ROC-AUC of 0.816. Interestingly, negative emotions such as fear, anger, and disgust were more influential for the prediction than were positive emotions such as joy (Fig. 1). These findings indicate that the framing of content regarding sentiment and emotional responses, rather than the actual information content, is pivotal to predicting engagement. The Source user features, including the number of followers, the number of statuses and tokens in the user's description, also contributed to the prediction (Figure S3).

### Sentiment dynamics

To better understand the transformation of content between Source and Quote, we examined each Source–Quote pair's sentiment change toward Israel. We scored each Source and Quote between − 1 and 1, and classified Sources into five significantly different sentiment groups toward Israel based on the sentiment score of the paired Quote (Kolmogorov–Smirnov, *p* value < 0.05) as follows: (1) strong oppose [− 1,− 0.5], (2) weak oppose [− 0.5,0), (3) neutral, (4) weak support (0,0.5], (5) strong support [0.5,1].

**Figure 3:**
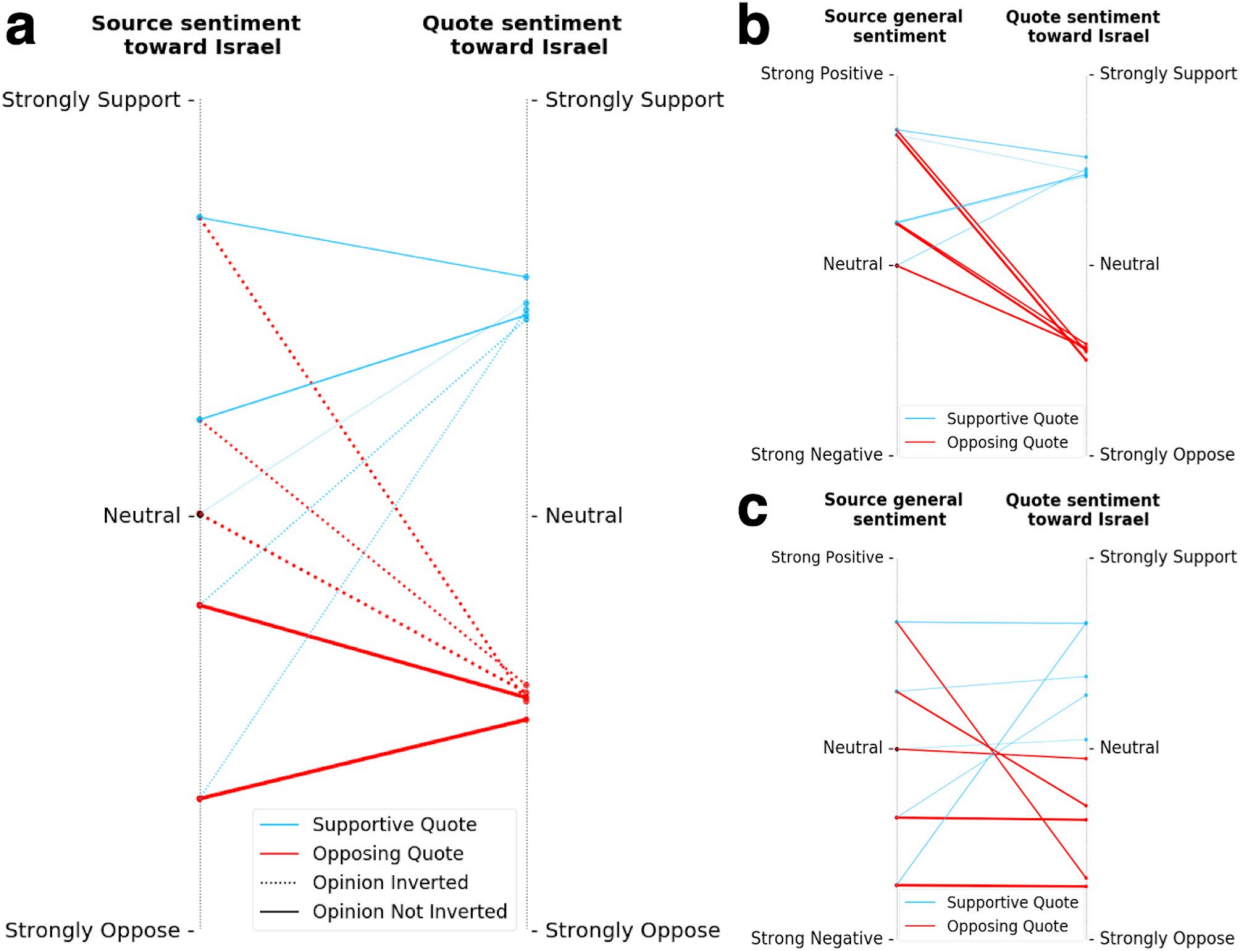
Source–Quote pair analysis. (**a**) Sentiment change toward Israel between the Source and the paired Quote for the 6174 relevant pairs. Each line represents a Source–Quote pair. The left-side position of each line indicates the average sentiment toward Israel expressed in the Source Tweets, and the right-side position of a line indicates the average sentiment toward Israel expressed in the paired Quote Tweet. The Quote polarity toward Israel determines the color of each line. Dotted lines represent sets that have experienced O.I. The thickness of each line indicates its volume. (**b**) Sentiment change between the Source and the paired Quote for the 6174 relevant pairs. Here, we compare the general sentiment (NLTK value) of the Source and the paired Quote sentiment toward Israel. (**c**) Sentiment change between the Source and the paired Quote for the 973 irrelevant pairs. Here, we compare the general sentiment (NLTK value) of the unrelated to domain Source and the paired Quote sentiment toward Israel.

We found that the probability of a Source undergoes O.I. depends on its sentiment toward Israel; the more supportive the Source, the higher its probability of experiencing O.I. (reflected by thicker lines in Fig. 3a). For example, Sources with a strong support sentiment for Israel were 3.0 times more likely to undergo O.I. than Sources with strong oppos toward Israel, 0.63 vs. 0.21 (see Table S10).

We also found in Sources that underwent O.I. that the Quotes sentiment levels toward Israel were similar (*t* test, *p* value > 0.05) (Fig. 3a). For example, considering Sources with a strongly oppose or a weakly oppose sentiment toward Israel that underwent O.I., their Quotes' sentiment levels toward Israel have, on average, the same magnitude. This trend remains significant when we examined the Source's general sentiment regardless of its sentiment towards Israel (Fig. 3b). For example, for Sources with strong or weak positive sentiments that underwent O.I., their Quotes' sentiment levels toward Israel have, on average, the same magnitude.

As high as 14% of the pairs explored included Quotes that were related to our domain while their Sources were unrelated to the domain. For example, the Source reported a favorable outcome of a baseball game, while the Quote suggested: "Palestine might have a team if 30 bombs hadn't killed 90 of them after one pissant IED attack killed 3 people. https://bit.ly/394tVAH". In contrast to our previous findings, we found that the Quote's magnitude of sentiment toward Israel maintains, on average, its Source general sentiment magnitude (Fig. 3c). For instance, Quotes of Sources with a strong positive general sentiment exhibit strongly support or strongly oppose sentiments toward Israel. Likewise, Quotes of Sources with a weak positive general sentiment maintain weak support or oppose sentiments toward Israel.

## Discussion

We explored the Opinion Inversion (O.I.) phenomenon, using politically-oriented discourse related to Israel. We showed that the transformation of Tweet content is highly common and can be predicted. Because political debates worldwide are generally highly emotional, predicting which Source will undergo O.I is possible with no need to understand the content. Using large-scale data from Twitter about debate related to Israel, we showed that the sentiment of a message and the emotions it triggers in the reader—and not the actual message—explain over 90% of the information gained for the prediction.

We found that as high as 14% of the pairs explored included Quotes that were related to our domain while their Sources were unrelated to the domain. This phenomenon can be partly related to online trolling, which is widespread on social media^55^. The online trolling in political discourse aims to promote political agenda using extreme statements, to elaborate a conflict^55–57^. Additionally, as pairs of source–Quote typically account for a high volume of engagement (i.e., retweets and likes), an observation which is in line with a previous work^37^, part of the Quotes are likely posted strategically to maximize engagement. Future studies could evaluate the potential benefit of an out-of-context O.I.

Our analysis is based on data related to political debates concerning Israel, and similar studies may reveal different patterns in other political contexts. For example, while we found that the probability that a Quote text contradicted its Source is 0.13, Garimella et al.^37^ found that only 0.042 of Quotes in a different context disagree with their Sources. Nevertheless, given the generality of our findings and the observation that sentiment and emotions in text serve as powerful indicators for the prediction of engagement^26,30,33^, we expect that our findings will be broadly applicable.

We found that sentiment and strong emotions serve as predictors of O.I. rather than drivers for O.I. Specifically, by solely accounting for content features, our model achieved an ROC-AUC of ~ 0.82. These findings are inline with previous studies that suggested that sentiment and emotions drive virality^26,29–33^. Nevertheless, sentiment and strong emotions may serve as confounding factors for O.I.'s actual drivers for a high degree of engagement.

We did not explicitly consider the structure of the network or the time elapsed between the sources and Quotes to model the diffusion of engagement with content. Interestingly, a recent study indicated that while Twitter users are typically exposed to political opinions that agree with their own^58^, there are users who try to bridge the echo chambers, and these users have to pay a "price of bipartisanship" in terms of their network centrality^59^. Our analysis further indicated that content with a strong-support toward Israel has a high probability of being inverted. Thus, it may be better for Israel's supporters to use content with a weaker support sentiment. The same logic also applies to opposers of Israel. Thus, future studies can model opinion diffusion on social networks that explicitly considers the O.I. phenomenon.

The sentiment polarity model was trained based on Tweets that were labeled by Israeli students, which might not accurately reflect the sentiment polarity of non-Israelis. Nevertheless, we chose native English speakers’ students, who lived for more than six months abroad. In addition, we supplied the students with a coding scheme and supporting examples for correct labeling. Notably, the vast majority of the Tweets are very straightforward to label, and particularly those that received high attention. Thus, we believe that potential biases arising from the labeling procedure are unlikely to affect our key findings.

In short, accounting for the transformation of contents in social networks is pivotal for the determination of strategies to increase exposure in political discourse. In practice, predicting O.I. can be achieved automatically and in real-time, with no need to understand the actual content of a message. Thus, our work contributes to understanding propagation, transformation, and dissemination of content and sentiment in social networks.

## Supplementary Information

Supplementary Information.

## Data Availability

The datasets generated during the current study are not publicly available due to Twitter's Developer Agreement but are available from the corresponding author on reasonable request.
