# Supplementary material for: Using sentiment analysis to predict opinion inversion in Tweets of political communication
Source: Sci Rep. 2021 Mar 31;11:7250. doi: 10.1038/s41598-021-86510-w (PMC8012385; doi:10.1038/s41598-021-86510-w)
Supplement: Supplementary file 1 — Supplementary Information. [file 41598_2021_86510_MOESM1_ESM.pdf]

Supplementary Information: Using sentiment analysis to predict opinion inversion in Tweets of political communication

Yogev Matalon, Ofir Magdaci, Adam Almozlino, Dan Yamin*

Table of Contents

S.1. Data collection **2**
S.2. Relevance classification model **4**
S.3. Polarity toward Israel classification model **8**
S.4. Source-Quote Pair prediction of sentiment polarity toward Israel **13**
S.5. Model comparison for prediction of Opinion Inversion **14**
S.6. Opinion Inversion model – Feature importance **14**
S.7. Opinion Inversion model's features **15**
S.8. Sentiment methods' comparison **16**
S.9. Labeling schema **17**
S.10. Source-Quote transitions **19**
References ***20***

S.1. Data collection

Phase 1: we randomly extracted English language Tweets related to Israel posted between January 6, 2008 and February 12, 2018 using Twitter API. To extract the content of the Tweets, we first scoped our example domain by creating a set of 30 general keywords and hashtags related to Israel with a focus on political content (Table S1). We then constructed a series of textual queries that selected Tweets for inclusion based on content.

Google search is the most popular service people use to search for information on today's Internet. Google search keywords, therefore, represent topics in which users are interested, and popular keywords represent hot trends, although the detailed mechanisms behind Google Trend are unknown. Search keywords have become a good indicator to understand activities in the real world[1]. To determine the most popular Israel-related keywords and events on Twitter, we searched various platforms, including Google Trend, Facebook, the Israel on Campus Coalition (ICC), and BDS reports.

We constructed two types of queries: 1) Subject-oriented queries, which contain hashtags and keywords that reference the example domain entities and topics without referencing a specific spatiotemporal event. Example hashtags and keywords are #BDS, #LoveIsrael, #StopTheWall, Israel, Gaza, #SupportIDF, #FreePalestine.

2) Spatiotemporal event-oriented queries, which contain hashtags and keywords that reference specific spatiotemporal events within the example domain. These include hashtags and keywords that trend during military operations or political and other events localized in space and time (e.g. the recent earthquakes in Nepal and Mexico, Israel Apartheid Week, UN conferences). Using these two types of queries, we were able to collect 715,894 Tweets.

Phase 2: We used these data to filter 7,147 Quotes by identifying Tweets that end with a link to another Tweet. Using Twitter API, we extracted the attributes for each Source and Quote (Table S2).

Table S1. *Set of keywords and hashtags related to Israel (The most 8 frequent queries).*

Query	% in corpus	% in Source-Quote pairs
Israel	62%	35%
Gaza	19%	4%
Palestine	17%	7%
Palestinians	6%	4%
#BDS	2%	3%
#SJP	5%	0.3%
#BoycottIsrael	0.3%	0.1%
#WorldWithoutWalls	0.03%	0.01%

The left column shows the word or hashtag which has been extracted, and the other columns represent the percentage of appearances in the full corpus and the Source-Quote pairs.

Table S2. *Data attributes.*

Tweet attributes	User attributes
id_str	screen_name
created_at	name
favorite_count	user_id_str
retweet_count	created_at
reply_count	profile_image_url
is_retweet	location
text	description
language	favourites_count
user_id_str	followers_count
screen_name	freind_count
urls	statuses_count
coordinates	utc_offset
media	verified
tweet type (Source/Quote)	
original_tweet_id_str	
original_tweet_text	
original_tweet_retweet_count	

The left column shows the Tweet attributes, and the right column shows the user attributes.

S.2. Relevance classification model

Using the content of Tweet, the model will determine whether the Tweet is relevant or related to the Israel domain. We define a relevant Tweet as one that addresses Israel or Israeli affairs (both political and non-political) or that addresses directly opposite entities in public opinion debate, such as BDS (Boycott Divestments, Sanctions), SJP (Students for Justice in Palestine), and so on. Non-relevant Tweets are defined as: (1) Tweets that contain some of the search keywords, but out of the case study context. For example, SJP could be used as an abbreviation for either 'Students for Justice in Palestine' or 'Sarah Jessica Parker'. (2) Tweets that may relate to Israel or to other entities in the domain but are not likely to shift public opinion toward Israel, such as those that reference the weather or sports results.

Out of the 716,000 Tweets, seven Israeli students **manually** labeled 4,841 random Tweets. The students were asked to make their judgment on what a layperson will most likely infer regarding the relevance of the Tweet to the domain based on the above case definition (see S9, Labeling schema).

Of the 4,841 labeled Tweets, 561 of them were irrelevant, while 4,280 were relevant. Due to the limited number of labeled Tweets and our preference to have misses (i.e. false negatives) rather than false alarms (false positives), we did not use downsampling or any other technique to balance the data. We allocated 4,342 Tweets as a training set and 499 Tweets as a test set (~10% test set). The training set and the test set were sampled randomly.

S2.1. Pre-process

Each Tweet that entered the pipeline went through the following steps:

a) Lower-case conversion to the entire Tweet.
b) Strip punctuation - in which we discard the following special characters: !"$%&()*+,./\:;<=>?[]^_`{|}~'-\#.
c) Tokenization - demarcating the Tweet (a string) into a list of tokens (words and substrings with lexical meaning).

d) Special content handling - this includes removing URLs and mentions (equivalent to user tagging in a post or comment, identified by using '@' sign, followed by user's screen name).

e) Hashtag handling - after inspecting the data, we noticed that some users incorporated hashtags as part of speech, i.e., using hashtags as regular words in sentences. For example: "#AIPAC has finally met its match with #SJP on campuses". However, some users use hashtags as tags, an indicator of the Tweet subject, opinion, or purpose. For example: "Opinion: Condemnation of Israel's Actions: It's Anti-Semitism, Not Criticism - Jewish Exponent #Israel #Gaza". We, therefore, kept each hashtag as is, and also duplicated each hashtag into a regular word (without the hashtag sign, which was removed in step b.). Because the users' writing preferences vary, this method allows us to consider the importance of keywords, as well as the unique behavior of hashtags.

f) Stop-word removal - we removed stop-words using the NLTK Python package [2] which holds a list of all stop-words existing in the English language. This step was skipped for a group of saved words (S2.2) we defined.

g) Slang correction - we created a small dictionary of common slang words and their meanings. Some examples are: 'imo'->'in my opinion', 'bc'->'because', 'fyo'-> 'for your information', etc.

h) Stemming - stemming was done by adjusted Porter stemmer. Each token is stemmed by Porter stemmer unless it belongs to a small group of words that required dedicated stemming, both for our convenience and avoiding merging words with different meanings to the same stem. One example of this dedicated stemming is: "Israelis" ->" Israeli" (instead of "Isra"). This step was skipped for a group of saved words such as names of countries, cities, organizations, politicians, news companies (e.g. BBC), and keywords of interest.

S2.2. Feature engineering

The algorithm accounts for a few main types of features:

- Basic features – number of characters, number of words, number of capital letters, etc.
- Hashtags and mentions features – a count of the number of mentions and hashtags in the Tweet. We also added Boolean features indicating whether a

common hashtag, such as #boycottisrael and #standwithisrael, appears in the Tweet.

- Tweet content and media – Boolean features indicate whether the Tweet contains a link, a request for retweet, or a photo embedded in it.
- Word tagging – counts the types of words in the text (e.g. noun, verb, adjective, adverb, coordinating conjunction, etc.). These features utilize NLTK Python package.
- Bag of words features - we developed both binary and counter features for the frequency of the top 100 words, selected by maximizing information gain (entropy reduction). Information gain was calculated only for words that appeared at least 4 times in the training set.
- Entity features – a family of features, indicating whether there are domain entities in the Tweet, and the identity of those entities. Possible entities include terrorist organizations, cities, and politicians' names.

S2.3. Feature selection

Feature selection was done in two steps: (1) Filtering zero-variance features and (2) filtering features with the lowest 25% feature importance, using a Python Scikit-learn package Random Forest model[3]. The most important features were the number of dominant words and appearances of domain words and hashtags.

S2.4. Model comparison

We compared several classification models on the training set, using 5-fold cross-validation:

1. Naïve Bayes classifier[4]. Priors were fit using the data distribution: 0.116 as irrelevant, and 0.884 for relevant Tweet.
2. Support Vector Machine (SVM) [5]. The data were scaled using Z-standardization. Polynomial kernel was tested with a variety of tuned parameters. We performed a grid search with 5-fold cross-validation. Parameters have been chosen to maximize the ROC AUC: C=0.1, degree=3, gamma=1

3. Logistic Regression [3]. The data were scaled using Z-standardization. Tuned parameters were regularization hyper-parameters. Parameters were chosen to maximize the ROC AUC: C=1, penalty=L2
4. Random Forest [6]. We performed a grid search with 5-fold cross-validation to select the number of trees as well as their depth, and feature selection criteria. Parameters were chosen to maximize the ROC AUC: Criterion=entropy, Max depth=20, N estimators=250, Min samples split=5

All models were implemented in the Scikit-Learn [3] python package. Based on the AUC train results, the selected model was Random Forest (Figure S1).

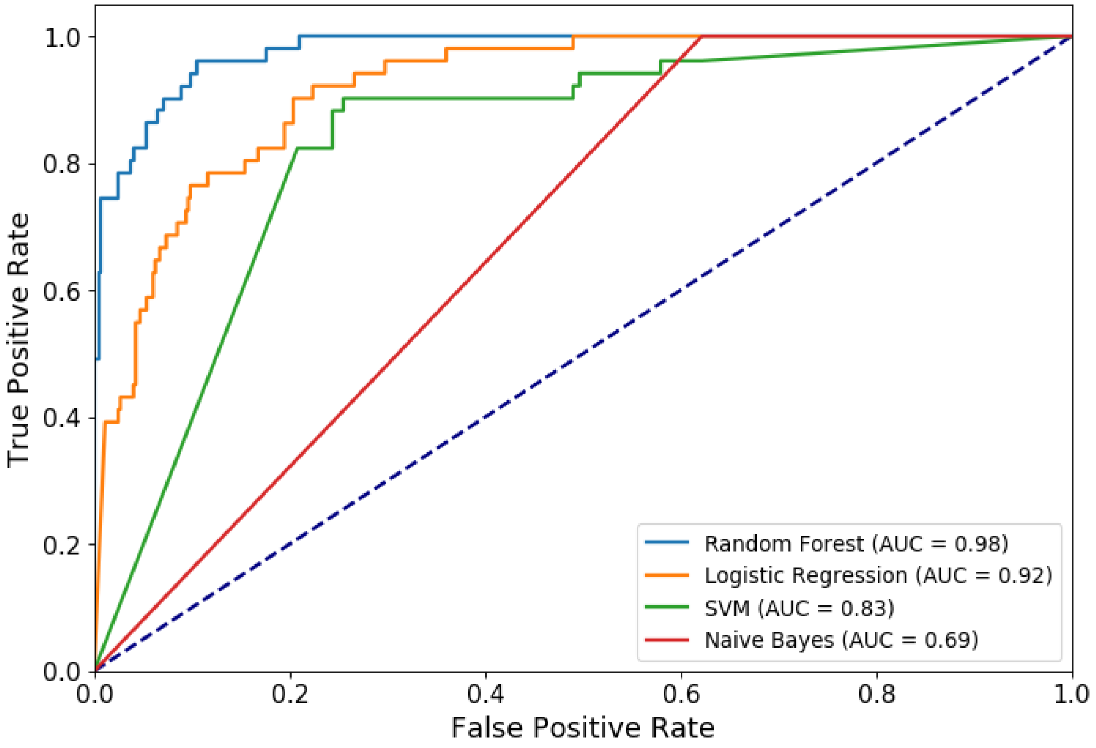

Figure S1. *ROC curve of the relevance algorithm for each trained model.*

S2.5. Model evaluation

The algorithm was able to reach 96% accuracy (Table S3) and an ROC AUC of 0.98 on the test set.

Benchmarking: despite the unbalanced priors (~88% of the labeled Tweets are relevant), the algorithm performance reflects significant improvement from the naïve policy of classifying Tweets using the major label.

Table S3. *Confusion matrix of the test set for the relevance model.*

	**Relevant prediction**	**Irrelevant prediction**
**Relevant label**	440	7
**Irrelevant label**	13	38

The rows identify the true label, and the columns identify the model's prediction.

S.3. Polarity toward Israel classification model

The polarity toward Israel model takes as input a relevant Tweet (see S.2, Relevance classification model) and determines whether the Tweet displays antagonism to Israel- Opposing (-1) or reveals sympathy to Israel – Supporting (+1), or is neutral— namely, not supporting and not opposing (0), Each category represents the side of the Tweet in the political debate. For example, the Positive category includes Tweets that revealed sympathy to Israel or opposed the other side. The classification algorithm was obtained by manually labeling 4,500 Tweets by 7 Israeli students. The students were asked to make their judgment on what a layperson will most likely infer from the Tweet concerning Israel- Supportive, Neutral, or Opposing (see S9, Labeling schema).

0.44 of these were labeled as having negative sentiment toward Israel, 0.3 were neutral, and 0.26 supported Israel. To evaluate the tagging process, we used a kappa coefficient (Cohen, 1960). The kappa statistic value for 100 Tweets was 0.8, which is substantial when comparing to prior studies [7].

The training set (90%) and the test set (10%) were sampled randomly. Each Tweet was pre-processed as for the relevance model (S2.1).

S3.1. Feature engineering

The algorithm utilizes all the features that were added for the relevance algorithm (S2.2), along with several additional types of features:

1. Sentiment and objectivity – We determined the sentiment of each Tweet using NLTK Python open-source sentiment analysis[2]. This algorithm specifies sentiment and objectivity score. The text sentiment is expressed as a real number in the range [-1.0, 1.0], which indicates the general intent identified in the Tweet

(-1: negative, and 1: positive). Subjectivity is expressed as a real number in the range [0.0, 1.0] where 0.0 is very objective and 1.0 is very subjective.

2. Emotions – we utilized the IBM Watson Tone Analyzer service [8], to define the following 13 emotion and emotional related characteristics for each Tweet:
    a. Basic emotions - anger, disgust, fear, joy, and sadness.
    b. Analytical - reasoning and analytical attitude about subjects
    c. Confidence - degree of certainty
    d. Tentativeness - degree of inhibition
    e. Openness - the level of which the author is open to experience a variety of activities
    f. Conscientiousness - a tendency to act in an organized or thoughtful way
    g. Extraversion - a tendency to seek stimulation in the company of others
    h. Agreeableness - tendency to be compassionate and cooperative towards others
    i. Emotional Range - sensitivity to the environment
3. User bio – we analyzed each user's description as presented on his or her profile page. Using the bag-of-words approach, we searched the descriptions for keywords that may indicate the user's political leaning. Some keywords that may be associated with pro-Israel users are: 'Jew', 'pro-Israel', etc. However, anti-Israel user descriptions may contain words such as 'Gaza', 'jihad' and 'apartheid'.
4. Time and event features – We constructed an event log containing roughly 200 political events regarding Israel between January 2008 and January 2018. These events include wars, terror attacks, peace negotiations, military operations, a major vote, speech or debate scheduled in the United Nations and elsewhere. Using this documentation, we were able to determine whether a Tweet was published during an event, and if so, the event's type and how many days have passed since it began.
5. Country support – because many Tweets refer to third-party countries, such as Russia, the United States, the United Kingdom, and more, we developed a dedicated feature to consider the impact of Tweets mentioning third-party countries. Based on the Anti-Defamation League Global 100 anti-Semitic survey (http://global100.adl.org/about), we created an 'Israel support index',

representing the anti-Semitic level of each country. In cases where several countries are mentioned in the Tweet, we used the min, max, average and median support.

6. Bag of words features - we developed both binary and counter features for the frequency of the top 125 words, selected by maximizing information gain (entropy reduction). Information gain was calculated only for words that appeared at least 20 times in the train set.
7. Sentiment score multiplied by the value of each dominant word.

S3.2. Feature selection

Feature selection was carried out by filtering features with the lowest 70% feature importance, using a Python Scikit-learn Random Forest model[3].

S3.3. Model comparison

We compared several classification models on the training set, using 5-fold cross-validation.

3 All-Versus-One model for each label- Our prediction will be determined by the class with the highest probability:

1. Naïve Bayes classifier [4]. The data were scaled using Z-standardization. Priors were fit using the following data distribution: 0.44 for a negative Tweet, 0.3 for a neutral Tweet, and 0.26 for a positive Tweet.
2. Support Vector Machine (SVM) [5]. The data were scaled using Z-standardization. The polynomial kernel was tested with a variety of tuned parameters. We performed a grid search with 5-fold cross-validation. Parameters were chosen to maximize the ROC AUC of each sub-model.
3. Logistic Regression [3]. The data were scaled using Z-standardization. Tuned parameters were regularization hyper-parameters. Parameters were chosen to maximize the AUC of each sub-model.
4. Random Forest [6]. We performed a grid search with 5-fold cross-validation for selecting the number of trees, their depth, the feature selection criteria, and so on. Each model was trained to maximize the AUC of each sub-model.

Multiclass classification:

5. XGBoost[9]. We performed a grid search with 5-fold cross-validation for selecting the number of trees, the learning rate, the sampling ratios, and so on. Each model was trained to maximize accuracy.
6. LightGBM[10]. We performed a grid search with 5-fold cross-validation for selecting the number of trees, their depth, the bagging configurations, and so on. Each model was trained to maximize accuracy.
7. CNN-LSTM[11]. Convolution Neural Network (CNN) with long short-term memory (LSTM) structure. We generated a 1D convolutional layer with 32 filters (ReLU as activation function) followed by a max-pooling layer. The output was then flattened to feed into LSTM layers. The model has 1 hidden LSTM layer (with 100 nodes) followed by a dense layer to provide the output. The output has 3 nodes with a SoftMax function and indicates the probability for each label. The input for the network is an embedding[12] layer with a vocabulary size of 8812 and an input length of 18 (Figure S2.C). The architecture was chosen by minimizing the Akaike information criterion (AIC), where the likelihood was defined to be the cross-entropy function. The cross-entropy measure was adopted because it was previously identified as a better means of measuring network performance in terms of error [13].

All models were implemented in Scikit-Learn [3] and Keras [14] Python packages. Based on the accuracy test results (Figure S2.A), the Random Forest model was selected for further analyses.

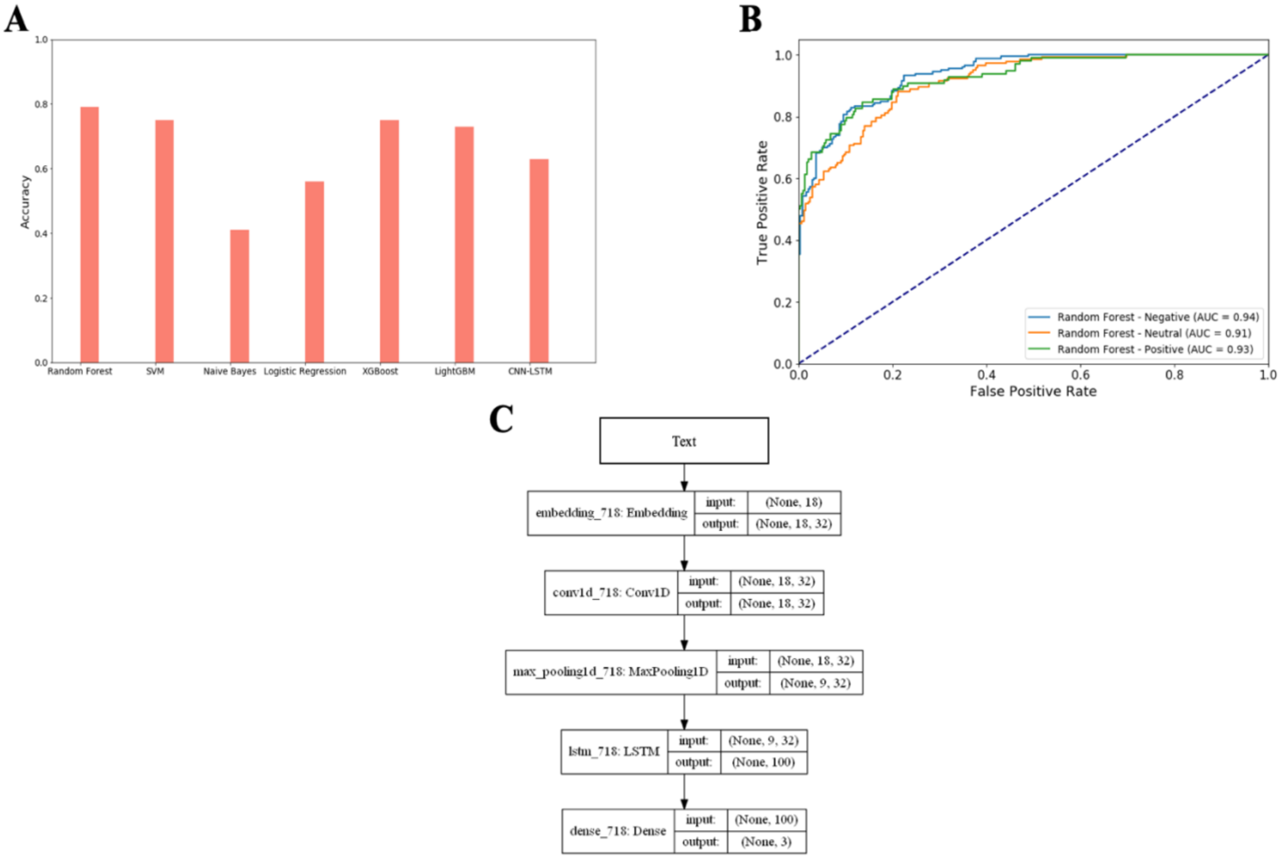

Figure S2. *Classification models of sentiment polarity toward Israel. (A) Accuracy comparison of the sentiment polarity prediction for each tested model. (B) ROC curve of the sentiment polarity algorithm for each sub-model of the Random Forest. The AUC value is shown in the legend. (C) CNN-LSTM model structure.*

S3.4. Model evaluation

The algorithm was able to reach 79% accuracy (Table S4) and an ROC AUC of 0.94, 0.91, 0.93 on the test set for the different labels (Figure S2.B).

Benchmarking: The algorithm performance reflects significant improvement from the standard sentiment analysis methods; 0.41 with NLTK and 0.31 with TextBlob python package [15].

Table S4. *Confusion matrix of the test set for the sentiment polarity prediction model.*

	**(-1) prediction**	**(0) prediction**	**(+1) prediction**
**(-1) label**	165	12	3
**(0) label**	34	100	9
**(+1) label**	21	10	67

The rows identify the true label, and the columns identify the model's prediction.

S.4. Source-Quote Pair prediction of sentiment polarity toward Israel

Table S5. *Source-Quote Pair prediction of sentiment polarity toward Israel.*

	**Source prediction**	**Quote prediction**	**Total prediction**
**Opposing (-1)**	3398	5671	9069
**Neutral (0)**	1494	1084	2578
**Supportive (+1)**	853	1176	2029

Each Tweet was classified by Sentiment polarity toward Israel.

S.5. Model comparison for prediction of Opinion Inversion

Table S6. *Comparison of model predictions of whether a message will undergo OI.*

Model	AUC	Accuracy	F1 score
Random Forest	0.835	0.796	0.82
XGBoost	0.832	0.793	0.81
Logistic Regression	0.791	0.782	0.81
Neural Network	0.711	0.629	0.59

S.6. Opinion Inversion model – Feature importance

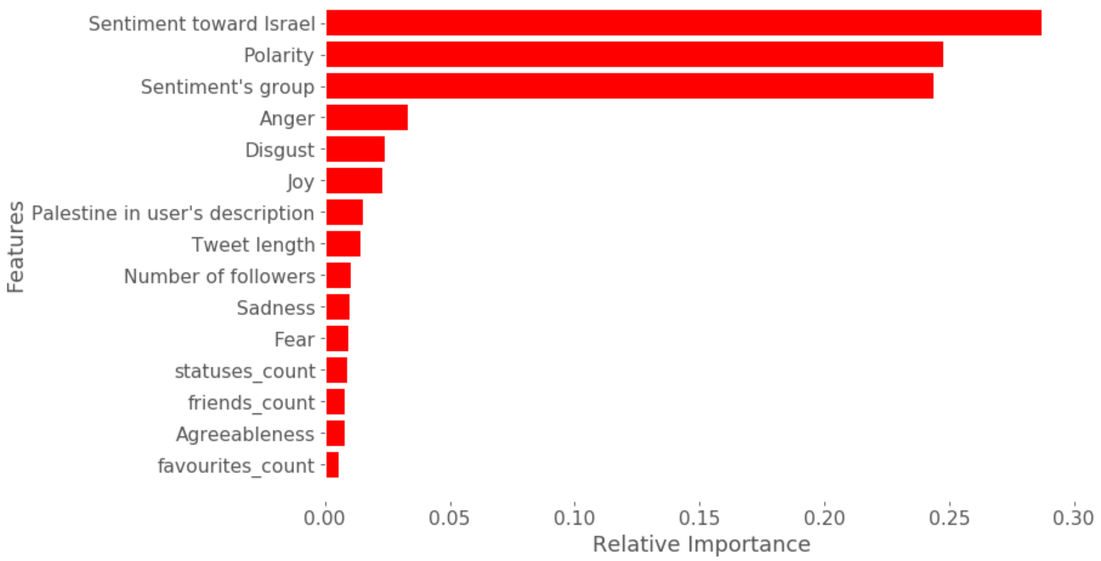

Figure S3. *Feature importance of the OI prediction model. Relative importance was calculated by averaging the decrease in impurity over trees, considering the interactions between all the features. Limited to the 15 most significant features. According to Scikit-learn Random Forest.*

S.7. <u>Opinion Inversion model's features</u>

Table S7. *Features of the OI prediction model.*

Content	User profile	User activity
Sentiment polarity toward Israel	Friend count	Favorite count
Sentiment toward Israel	Follower count	Status count
Sentiment group	Verified	
Disgust	11 Description's tokens:<br>1. Activist<br>2. News<br>3. Palestine<br>4. Jewish<br>5. Anti<br>6. Liber<br>7. Polit<br>8. Pro<br>9. Justice<br>10. Israel<br>11. freedom	
Anger		
Sadness		
Joy		
Fear		
Analytical		
Extraversion		
Openness		
Tentative		
Conscientiousness		
Agreeableness		
Confidence		
Emotional range		
Number of mentions		
Number of hashtags		
Media		

The features are categorized into three groups: content, user profile, and user activity.

S.8. Sentiment methods' comparison

Given the fact that different sentiment analysis tools can vary in their results, we checked the robustness of NLTK by comparing our results to SentiStrength method. First, we examined the degree of agreement between the two methods in the content group classification, and we found that in 75.3% of the Tweets, methods agreed on the content's group (Table S8).

Table S8. *Contents group prediction's comparison*

**VADER** **SentiStrength**	Strong Oppose	Weakly Oppose	Neutral	Weak Support	Strongly support
Strong Oppose	1590	621	0	0	0
Weakly Oppose	1782	4179	0	0	0
Neutral	0	0	2367	0	0
Weak Support	0	0	0	787	484
Strongly support	0	0	0	117	423

Second, we tagged the content's group of 500 pairs and found that the most accurate method was VADER with an accuracy of 80.2% (Table S9).

Table S9. *Methods' accuracy of content's group classification.*

**Method**	**Accuracy**
VADER	0.802
SentiStrength	0.778

In the prediction of which original Source Tweets will undergo OI, the sentiment toward Israel by SentiStrength still contributed the most to the prediction, accounting for 77% of the information gained (80% for VADER).

The same as VADER, using SentiStrength we showed that a Source with strong positive sentiment has a higher probability to undergo OI than does a Source with

weak positive sentiment. We also found that SentiStrength tends to classify weak sentiment more frequently than VADER. The difference between the content's classification affects the results of the strategic content framing. While VADER's strong sentiment Source was found to be significantly the most viral content, here weak content Source is more viral with no significant difference than strong content.

S.9. Labeling schema

In order to ensure our labeling process is not biased, we created a coding schema for the students who tagged the data. First, we chose native English speakers' students, who lived for more than six months abroad.

Second, we randomly split the 5,000 samples between them (~800 Tweets per student). To validate the consistency of the labeling for all students, we randomly chose 100 Tweets that all seven students labeled.

Third, we supply the following instructions and examples:

1. For each Tweet, label the "Relevance" of the Tweet (0 – irrelevant, 1 – relevant)
2. A Tweet is relevant if it includes any content linked to Israel, excluding weather and sports terms.
3. If the Tweet is relevant, label the "Sentiment Polarity" of the Tweet (Opposing, Neutral, Supporting). If the Tweet is irrelevant, please skip to the next Tweet.
4. How to label "Sentiment Polarity":
    a. According to your knowledge of the American culture, label as an average American reader.
    b. Opposing: The Tweet displays antagonism to Israel
    c. Supporting: Tweets that revealed sympathy to Israel or opposed the other side in the political debate
    d. Neural: Not supporting and not opposing, or the Tweet contains both of them
5. If you are not sure, please skip the problematic Tweet
6. Examples:

	Opposing (-1)	Neural (0)	Supporting (1)
Relevant (1)	#Dell considering R&D center in apartheid Israel: http://bit.ly/9HDt3t #BDS<br><br>Makes me sad every toy I see is made in #China. No wonder we don't have jobs in the US of Israel.<br>What would Mary & Joseph encounter in Bethlehem today? A 'security' wall built on illegally occupied land #Palestine<br><br>Pray for gaza pic.twitter.com/3ktPLA15Fb	Israel National News: Education Rabbi, Industrialist Plan Hareidi Vocationa<br><br>Native plants of #Palestine http://www...<br>Meanwhile, in Gaza, RT @washingtonpost: Israelis face a new round of psychological warfare using text messages<br><br>U.N agrees to recognise Palestine but UK abstains http://bit.ly/Va9frLÂ via @MailOnline The Islamic emirate of the UK. Sad.	After a trip day at the north of israel I can say that we have a nice country.<br><br>Ancient Well Discovered In Israel: Source: http://www.huffingtonpost.com<br>#Palestinians in the West Bank voted in #local elections on Saturday #different from Kenya's<br><br>France, Israel in solidarity over Toulouse shooting' - Jerusalem Post http://bit.ly/GIevxh
Irrelevant (0)	SJP : she'll never cease to #fashionspire us! harpersbazaarus's photo # http://instagram.com/p/crS9muw3Qf/Â<br><br>Watch Portugal U19 - Israel U19 Live 10/13/2012 http…		

We used the kappa coefficient over the 100 identical Tweets to validate the consistency between the students in the labeling process. The kappa statistic value for the "Relevance" labeling was 0.95, and 0.804 for the "Sentiment Polarity".

S.10. <u>Source-Quote transitions</u>

Table S10. *Transition probability matrix of the sentiment groups*

**Quote**<br>**Source**	Strong Oppose	Weakly Oppose	Neutral	Weak Support	Strongly support
Strong Oppose	0.426	0.398	0.083	0.043	0.05
Weakly Oppose	0.324	0.46	0.11	0.054	0.052
Neutral	0.281	0.377	0.178	0.075	0.089
Weak Support	0.222	0.328	0.133	0.152	0.165
Strongly support	0.282	0.346	0.094	0.105	0.173

References

1. Kwak, H., Lee, C., Park, H. & Moon, S. What is Twitter, a social network or a news media? in *Proceedings of the 19th International Conference on World Wide Web, WWW '10* 591–600 (2010). doi:10.1145/1772690.1772751
2. Bird, S., Klein, E. & Loper, E. *Natural Language Processing with Python: Analyzing Text with the Natural Language Toolkit*. *Text* (2009). doi:10.1097/00004770-200204000-00018
3. Pedregosa, F. *et al.* Scikit-learn: Machine learning in Python. *J. Mach. Learn. Res.* **12**, 2825–2830 (2011).
4. Rish, I. An Empirical Study of the Naïve Bayes Classifier An empirical study of the naive Bayes classifier. (2014).
5. Suykens, J. A. K. & Vandewalle, J. Least squares support vector machine classifiers. *Neural Process. Lett.* **9**, 293–300 (1999).
6. Breiman L. Machine Learning, 45(1), 5–32. *Stat. Dep. Univ. California, Berkeley, CA 94720.* (2001). doi:10.1023/A:1010933404324
7. Landis, J. R. & Koch, G. G. The Measurement of Observer Agreement for Categorical Data. *Biometrics* **33**, 159 (1977).
8. IBM. Watson Tone Analyzer service. Available at: https://console.bluemix.net/docs/services/tone-analyzer/index.html#about.
9. Chen, T. & Guestrin, C. XGBoost: A scalable tree boosting system. in *Proceedings of the ACM SIGKDD International Conference on Knowledge Discovery and Data Mining* **13-17-Augus**, 785–794 (Association for Computing Machinery, 2016).
10. Ke, G. *et al.* LightGBM: A highly efficient gradient boosting decision tree. in *Advances in Neural Information Processing Systems* **2017-Decem**, 3147–3155 (2017).
11. Sainath, T. N., Vinyals, O., Senior, A. & Sak, H. Convolutional, Long Short-Term Memory, fully connected Deep Neural Networks. in *ICASSP, IEEE International Conference on Acoustics, Speech and Signal Processing - Proceedings* **2015-Augus**, 4580–4584 (Institute of Electrical and Electronics Engineers Inc., 2015).
12. Mikolov, T., Sutskever, I., Chen, K., Corrado, G. & Dean, J. Distributed representations ofwords and phrases and their compositionality. in *Advances in*

Neural Information Processing Systems (2013).

13. Nasr, G. E., Badr, E. A. & Joun, C. Cross Entropy Error Function in Neural Networks: Forecasting Gasoline Demand. in *FLAIRS Conference* 381–384 (2002).
14. Chollet, F. Keras. (2015). Available at: https://github.com/fchollet/keras.
15. Loria, S. *Textblob Documentation*. (2018).
